# Dyskerin and telomerase RNA component are sex-differentially associated with outcomes and Sunitinib response in patients with clear cell renal cell carcinoma

**DOI:** 10.1186/s13293-023-00526-7

**Published:** 2023-07-11

**Authors:** Huiyang Yuan, Xin Qin, Qingya Yang, Li Liu, Zhiqing Fang, Yidong Fan, Dawei Xu

**Affiliations:** 1 Department of Urology, Qilu Hospital of Shandong University, Jinan, 250012 China; 2 School of Nursing, Beijing University of Chinese Medicine, Beijing, 100191 China; 3 Department of Medicine, Division of Hematology, Bioclinicum and Center for Molecular Medicine, Karolinska Institute and Karolinska University Hospital Solna, 171 76 Stockholm, Sweden

**Keywords:** ccRCC, DKC1, Sex difference, Sunitinib, TERC, Telomerase

## Abstract

**Background:**

Clear cell renal cell carcinoma (ccRCC) displays sex-biased incidence, outcomes, molecular alterations and treatment efficacy; however, clinical managements are largely identical in male and female patients. Moreover, many biomarkers have been identified as predictors for ccRCC outcomes and response to therapeutic drugs, such as multitargeted tyrosine-kinase receptor (TKR) inhibitors, but little is known about their sex-specificity. Dyskerin (DKC1), encoded by the *DKC1* gene within Xq28, is a telomerase co-factor stabilizing telomerase RNA component (TERC) and overexpressed in various cancers. Here, we determined whether DKC1 and/or TERC affect ccRCC sex-differentially.

**Methods:**

DKC1 and TERC expression in primary ccRCC tumors was assessed using RNA sequencing and qPCR. DKC1 association with molecular alterations and overall or progression-free survival (OS or PFS) was analyzed in the TCGA cohort of ccRCC. The IMmotion 151 and 150 ccRCC cohorts were analyzed to evaluate impacts of DKC1 and TERC on Sunitinib response and PFS.

**Results:**

DKC1 and TERC expression was significantly upregulated in ccRCC tumors. High DKC1 expression predicts shorter PFS independently in female but not male patients. Tumors in the female DKC1-high group exhibited more frequent alterations in *PIK3CA*, *MYC* and *TP53* genes. Analyses of the IMmotion 151 ccRCC cohort treated with the TKR inhibitor Sunitinib showed that female patients in the DKC1-high group was significantly associated with lower response rates (*P* = 0.021) accompanied by markedly shortened PFS (6.1 vs 14.2 months, *P* = 0.004). DKC1 and TERC expression correlated positively with each other, and higher TERC expression predicted poor Sunitinib response (*P* = 0.031) and shorter PFS (*P* = 0.004), too. However, DKC1 rather than TERC acted as an independent predictor (*P* < 0.001, HR = 2.0, 95% CI 1.480–2.704). In male patients, DKC1 expression was associated with neither Sunitinib response (*P* = 0.131) nor PFS (*P* = 0.184), while higher TERC levels did not predict response rates. Similar results were obtained from the analysis of the Sunitinib-treated IMmotion 150 ccRCC patients.

**Conclusions:**

DKC1 serves as an independent female-specific predictor for survival and Sunitinib efficacy in ccRCC, which contribute to better understanding of the sex-biased ccRCC pathogenesis and improve personalized interventions of ccRCC.

**Supplementary Information:**

The online version contains supplementary material available at 10.1186/s13293-023-00526-7.

## Highlights

- The telomerase components DKC1 and TERC are identified to contribute to sex-biased molecular alterations, survival and treatment efficacy in ccRCC.
- Higher DKC1 and/or TERC expression predicts shorter survival and poor response to Sunitinib treatment only in female patients with ccRCC.
- High DKC1 expressing-tumors in females display enhanced oncogene activation and tumor suppressor inactivation.
- The findings have implications in precision oncology of ccRCC.

**Supplementary Information:**

The online version contains supplementary material available at 10.1186/s13293-023-00526-7.

## Plain language summary

Many types of cancer including clear cell renal cell carcinoma (ccRCC) are known to display sex-biased survival, genomic alterations and treatment efficacy; however, clinical managements are largely identical in male and female ccRCC patients. Many molecules have been identified as predictors for ccRCC survival and response to therapeutic drugs, such as multitargeted tyrosine-kinase receptor inhibitor Sunitinib, but little is known about their sex-specificity. Dyskerin (DKC1), encoded by the *DKC1* gene on X chromosome, is a telomerase co-factor stabilizing telomerase RNA component (TERC), whereas telomerase plays key roles in cancer development and progression. In this study, we observed increased DKC1 expression in ccRCC tumors. High DKC1 expression predicts shorter disease progression-free survival (PFS) in female but not male patients. Oncogene activation and tumor suppressor inactivation are more frequent in the female DKC1-high tumors. By analyzing two cohorts of ccRCC patients treated with Sunitinib, we showed that female patients in the DKC1-high group was significantly associated with lower response rates accompanied by markedly shortened PFS. DKC1 and TERC expression correlated positively with each other, and higher TERC expression predicted poor Sunitinib response and shorter PFS, too. However, DKC1 rather than TERC acted as an independent predictor. In male patients, DKC1 expression was associated with neither Sunitinib response nor PFS. Thus, DKC1 serves as a female-specific predictor for survival and Sunitinib response in ccRCC. Our findings are expected to improve personalized management of ccRCC.

**Supplementary Information:**

The online version contains supplementary material available at 10.1186/s13293-023-00526-7.

## Background

Clear cell renal cell carcinoma (ccRCC) is the major subtype of renal cell carcinoma (RCC) composed of approximately 75–80% of all RCCs, and its incidence has steadily increased over the last decades globally, contrasting with many other types of cancer exhibiting significantly reduced incidence [1–3]. Most (~ 70%) ccRCC patients are diagnosed at early stages with localized disease, and thus successfully resected [4], whereas remaining patients present with advanced ccRCC. In addition, approximately 30% of patients with localized ccRCC will eventually undergo recurrence or metastasis after nephrectomy [4]. For all those advanced and recurrent/metastatic ccRCCs, adjuvant therapies are required [4–6]. Because ccRCC is intrinsically insensitive to radio- and chemotherapy, several novel treatment strategies have been applied, among which is multitargeted tyrosine-kinase receptor (TKR) inhibitors [5, 6]. Sunitinib, approved by FDA for the first line treatment of metastatic ccRCC in 2006, is such an inhibitor that targets the vascular endothelial growth factor receptors (VEGFRs), platelet-derived growth factor receptors (PDGFRs), Fms-like tyrosine kinase 3 (FLT3), colony stimulating factor-1 receptor (CSF1R), and tyrosine-protein kinase receptor RET [6]. Since the clinical application of Sunitinib, the prognosis of advanced or metastatic ccRCCs has been significantly improved and most patients benefit from the treatment with longer progression-free survival (PFS), but a subset of patients lose response due to acquired Sunitinib resistance. Moreover, approximately 1/3 of ccRCCs exhibit intrinsic resistance to Sunitinib [5]. Thus, distinguishing Sunitinib responders from non-responders and insights into underlying resistance mechanisms are both biologically and clinically important. Towards these ends, many studies identified factors involved in drug resistance and developed multigene expression signatures to predict ccRCC outcomes and response to Sunitinib, and mutations in tumor suppressors (*TP53*, *PBRM1* and *BAP1*) have also been shown to contribute to poor response to Sunitinib [7–11]. However, all those predictors are still insufficient from accurate patient stratification. Searching for more reliable biomarkers or molecular tools for ccRCC prognosis and personalized interventions is, therefore, a highly unmet task.

Sex differences occur across human cancer, which include incidence, mortality, outcomes, molecular alterations, therapeutic efficacy, among others [12–15]. This is also the case for ccRCC [13, 16, 17]. The ratio of male and female ccRCC is in general > 2:1 [16, 18], and ccRCC lung metastasis is fivefold higher in male than female patients [16, 19]. The analysis of 2055 nephrectomized ccRCC patients unrevealed that female sex was significantly associated with favourable outcomes [17]. Differences in sex hormones and molecular alterations have been shown as mechanisms underlying sex-biased metastasis; however, it remains to be defined whether other factors or signalling pathways are involved. Moreover, little is known of whether male and female patients respond differentially to TKR inhibitors. No detailed investigations have been so far performed to rigorously address the sex impact on therapeutic efficacy of TKR inhibitors, such as Sunitinib. Although numerous biomarkers have been identified to predict ccRCC survival and Sunitinib response, it is unclear whether they are equally powerful to both sexes. A thorough elucidation of these issues will benefit to precision oncology of ccRCC and provide insights into sex-related ccRCC pathogenesis as well.

Dyskerin (DKC1), encoded by *Dyskeratosis congenita 1* gene, is an enzyme that catalyzes RNA pseudouridylation [20, 21]. The pseudouridine incorporation stabilizes RNA molecules [20]. One of important DKC1 targets is non-coding telomerase RNA component (TERC) that serves as an internal template for telomerase-mediated telomere elongation [21, 22]. Telomerase is a multi-unit complex consisting of the catalytic component telomerase reverse transcriptase (TERT), TERC, DKC1, and others [23]. DKC1 deficiency induces diminished TERC expression and telomerase activity, thereby leading to telomere shortening and subsequent telomere pathologies [21, 24, 25]. Telomerase, activated in up to 90% of human malignancies, is required for infinite proliferation of cancer cells by maintaining telomere length [26]. The evidence has also accumulated that telomerase exerts many biological activities beyond its telomere-lengthening function, thereby promoting cancer progression or aggressiveness and drug resistance [26–31]. For instance, telomerase confers resistance to FLT3 inhibitors in leukemic cells [29]. DKC1 as one of key telomerase components is overexpressed in many types of cancer [23, 32–41]. Moreover, DKC1 also exhibits other biological activities, including regulation of ribosome biogenesis and splicing events [20, 21, 33, 38, 39]. Interestingly, DKC1 has been shown as a prognostic factor in ccRCC and higher DKC1 expression is associated with significantly shorter patient survival [23, 32].

The *DKC1* gene is located on X chromosome (Xq28), and its expression is may differ between male and female individuals, which consequently leads to differential TERC expression and telomerase activity for telomere lengthening [42, 43]. These features, together with the prognostic effect of DKC1 on ccRCC survival [23, 32], promote us to determine whether the DKC1 function is sex-dependent or DKC1 expression differentially predicts outcomes between female and male patients with ccRCC. As described above, telomerase is involved in resistance to the specific RTK FLT3 inhibitor [29], and there thus exists the possibility that DKC1, as a telomerase factor, may play a part in ccRCC patient response to Sunitinib. The present study is designed to address the issues above.

## Materials and methods

### Tumors and their matched noncancerous renal tissues (NT) from ccRCC patients

Twenty patients with ccRCC were recruited. Tumors and their matched NTs were collected from these patients who underwent nephrectomy. All the specimens were stored in nitrogen tanks until use. In all tumors and NTs from 20 patients, the half of them (10) were used for RNA sequencing and remaining 10 for PCR analyses. Their clinical information was listed in Additional file 1: Tables S1 and S2. The study was approved by the Institutional review board of Qilu Hospital of Shandong University (#KYLL-2021(KS)-192) and the signed informed consent was obtained from all patients.

### RNA extraction, qPCR and RNA sequencing

RNA was extracted from primary tissues using a RNAfast2000 kit (Fastagen). cDNA was synthesized using a PrimeScript™ RT reagent Kit (TAKARA). qPCR was performed using SYBR Green of RT Master Mix (TAKARA) to determine mRNA levels of target genes based on 2(− ΔΔCT) values. β-Actin mRNA levels were used as the internal control for normalization of target gene expression. PCR primers were: DKC1: 5′-ATGGCGGATGCGGAAGTAAT-3′ (forward) and 5′-CCACTGAGACGTGTCCAACT-3′ (reverse). TERC: 5′-ACCCTAACTGAGAAGGGCGTA-3′ (forward) and 5′-AATGAACGGTGGAAGGCGG-3′ (reverse). β-actin: 5′-CATGTACGTTGCTATCCAGGC-3′ (forward) and 5′-CTCCTTAATGTCACGCACGAT-3′ (reverse).

Sequencing libraries were generated using NEBNext^R^ Ultra™ RNA Library Prep Kit (New England Biolabs) following manufacturer’s recommendations. RNA sequencing was carried out using Illumina HiSeq 4000 sequencer at Metware Biotechnology (Wuhan, China). Paired-end reads were quality controlled by Q30 and Cutadapt software (v 1.9.3) was used to remove low-quality reads and 3’ adaptor-trimming. Hisat2 (v 2.0.4) was further used to align clean reads from RNA sequencing, and sequencing depth and gene length were adjusted by Fragments Per Kilobase of transcript per Million fragments mapped.

### Data collection and processing of ccRCC tumors

The TCGA cohort of ccRCCs included 525 tumor samples with survival information available and 72 adjacent renal NTs [18]. Transcriptome, mutation, copy number alterations (CNAs) and clinical–pathological data were downloaded from https://gdc.cancer.gov/. mRNA data have been corrected and standardized for the batch effects and the log2 transformed RSEM data were used for mRNA analysis. DKC1 protein expression was obtained from Clinical Proteomic Tumor Analysis Consortium (CPTAC) (http://ualcan.path.uab.edu/index.html). ccRCC patients receiving Sunitinib treatments were contained in IMmotion150 [9, 44–46] and IMmotion151 trials [44, 47].

### Proliferation, cancer stemness, epithelial–mesenchymal transition (EMT) and angiogenesis signature analyses

ccRCC proliferation scores were estimated using expression levels of Ki-67 mRNA and cell cycle signature, respectively. Cancer stemness, EMT and angiogenesis signature scores were calculated based on single sample gene set enrichment analysis (ssGSEA) or as the median z-score of genes included in each signature for each sample. These gene signatures are as follow: Angiogenesis: VEGFA, KDR, ESM1, PECAM1, ANGPTL4 and CD34 [48]. Cell Cycle: CDK2, CDK4, CDK6, BUB1B, CCNE1, POLQ, AURKA, MKI67 and CCNB2 [11]. EMT: VIM, CDH2, FOXC2, SNAI1, SNAI2, TWIST1, FN1, ITGB6, MMP2, MMP3, MMP9, SOX10, GCS, CDH1, DSP and OCLN [49].

### Statistical analysis

All statistical analyses were carried out using R package version 4.0.5. Wilcox and K–W sum tests were used for analysis of differences between two groups and among multi groups, respectively. Survival analyses were made using log-rank test. The Survival and Survminer packages were employed to draw Kaplan–Meier survival curves for visualization of OS and PFS. Univariate and multivariate Cox regression analyses were used to determine the effect (HR and 95% CI) of DKC1 and TERC on OS and PFS. *P* < 0.05 were considered as statistically significant.

## Results

### Upregulation of DKC1 expression in ccRCC tumors

**Fig. 1:**
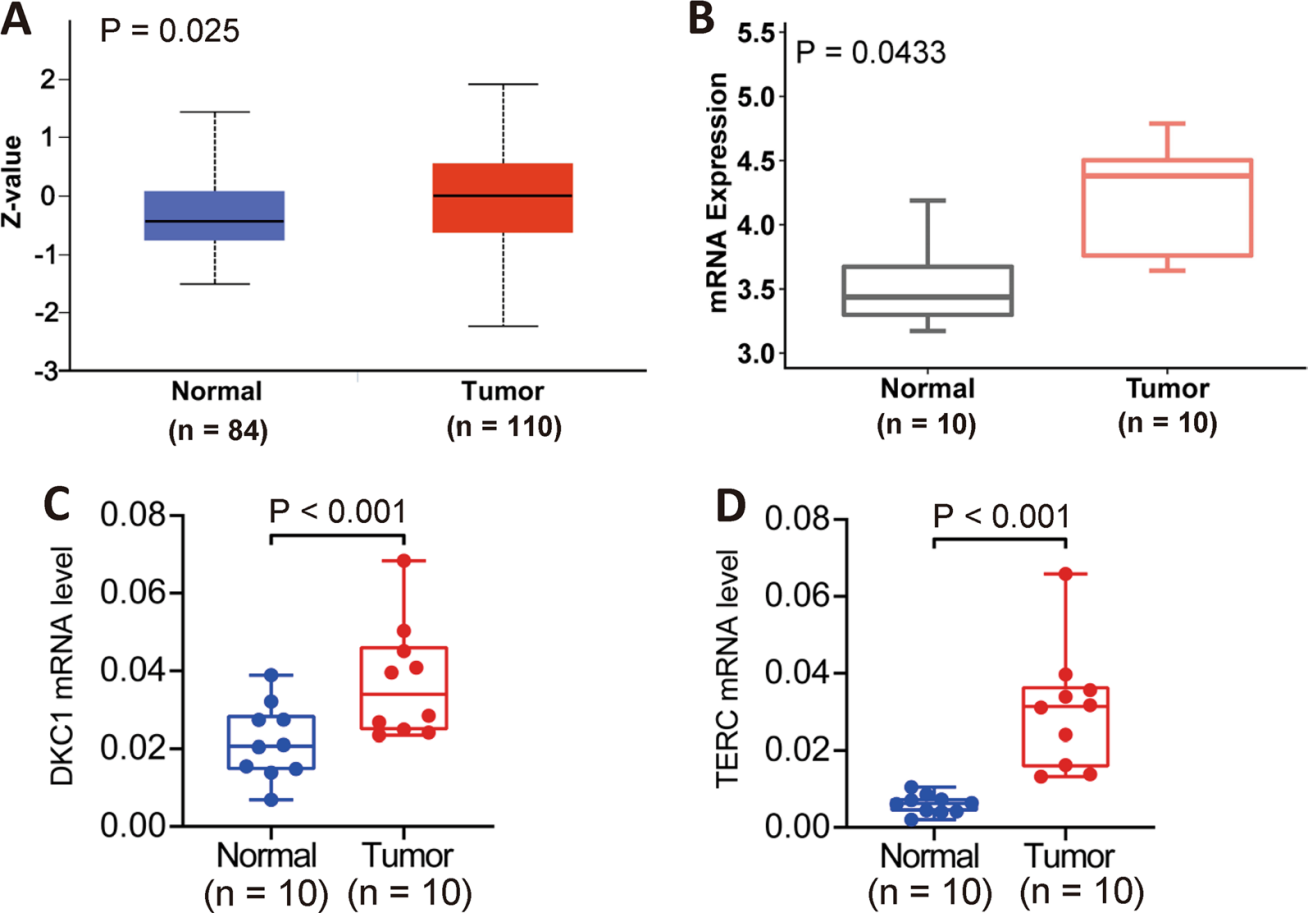
DKC1 and TERC expression is upregulated in ccRCC tumors. **A** Enhanced DKC1 protein expression in ccRCC tumors. The DKC1 expression data were obtained from Clinical Proteomic Tumor Analysis Consortium (CPTAC) (http://ualcan.path.uab.edu/index.html). Multiplexed quantitative mass spectrometry-based proteomic targeted assays were performed on 84 NTs and 110 ccRCC tumors from the CPTAC cohort. **B** Upregulation of DKC1 expression in primary ccRCC tumors as determined using RNA sequencing. Tumors and matched NT tissues from 10 ccRCC patients were subject to the transcriptomic analyses. DKC1 mRNA levels were expressed as PKMT. (**C** and **D**) Upregulation of both DKC1 and TERC expression in ccRCC tumors. Tumors and matched NT tissues from the additional cohort of 10 ccRCC patients were analyzed for their DKC1 and TERC transcripts using qPCR assay. **A** and **B** were analyzed using Wilcox sum test, while **C** and **D** were analyzed using Student T test

Our recent study has shown the upregulation of DKC1 mRNA expression in the TCGA cohort of ccRCC tumors [23]. Multiplexed quantitative mass spectrometry-based proteomic targeted assays further demonstrated significantly enhanced DKC1 expression at a protein level (Fig. 1A) (http://ualcan.path.uab.edu/index.html). To confirm the results above, we carried out RNA profiling on 10 primary ccRCC tumors and their matched nontumoral tissues (NTs), and significantly higher levels of DKC1 expression in tumors than in NTs were observed (Fig. 1B). In addition, DKC1 mRNA expression was determined in tumors and matched NTs from another cohort of 10 ccRCC patients using qPCR and similar results were obtained (Fig. 1C). TERC RNA was simultaneously assessed, and its overexpression also occurred in this cohort of ccRCC tumors (Fig. 1D).

### DKC1 as a survival predictor in female but not male ccRCC patients

**Fig. 2:**
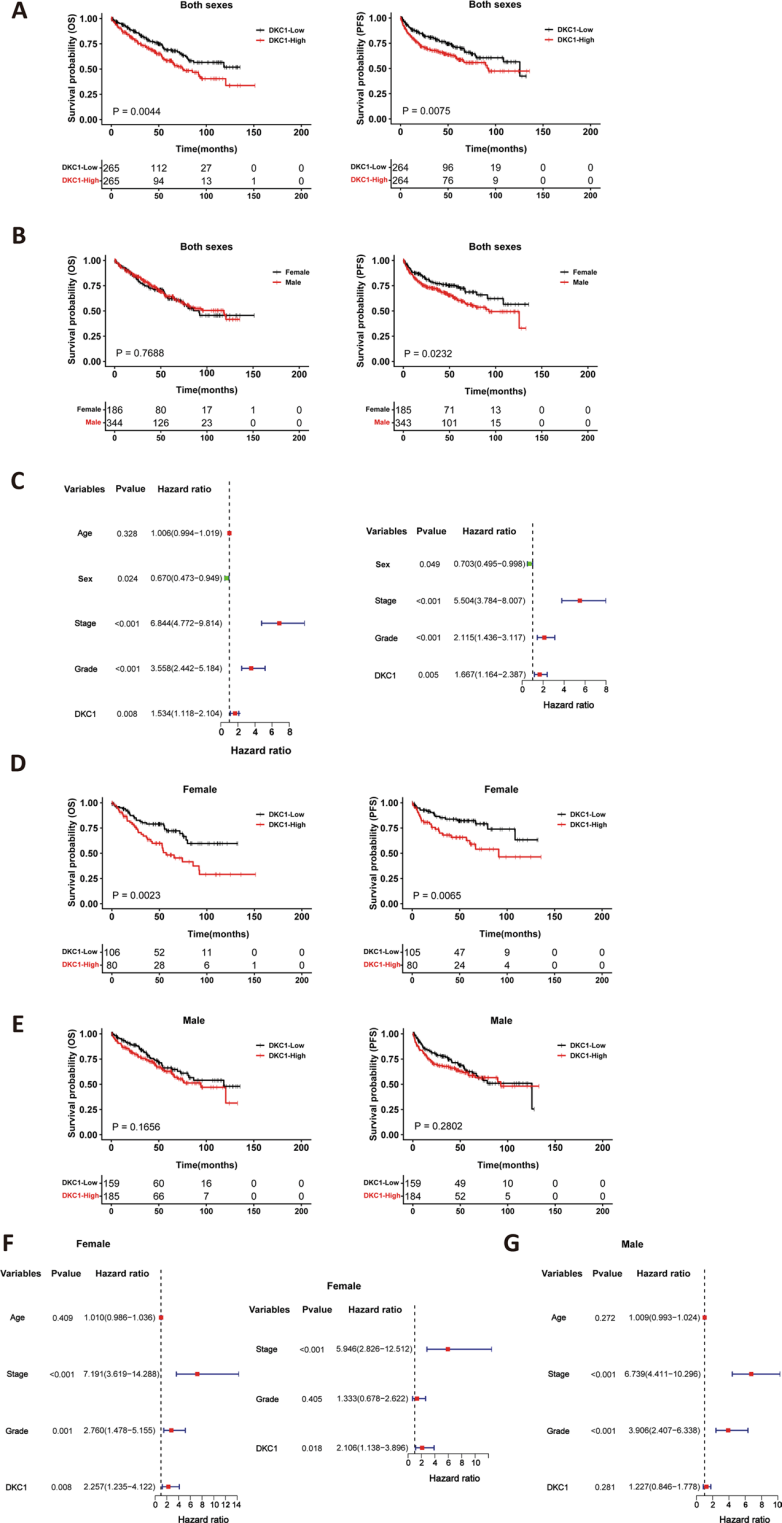
DKC1 expression is associated with overall and progression-free survival (OS and PFS) in female but not male patients with ccRCC. The analysis was performed on 530 patients in the TCGA ccRCC cohort. Patients were classified into DKC1-low and high groups using median DKC1 mRNA levels in tumors as a cutoff point. **A** Kaplan–Meier survival analyses of 530 ccRCC patients based on DKC1 levels (both sexes). Left: OS and right: PFS. **B** Kaplan–Meier survival analyses of 530 ccRCC patients based on sex. Left: OS and right: PFS. **C** Univariate (left) and multivariate (right) COX regression analyses based on age, sex, stages, grades and DKC1 expression. **D** Significant differences in OS and PFS between DKC1-low and high groups in female ccRCC patients. **E** Lack of association of DKC1 expression with OS or PFS in male ccRCC patients. **F** DKC1 as an independent prognostic factor as assessed using univariate (left) and multivariate (right) COX regression analyses. **G** Univariate COX regression analyses of DKC1 expression with survival association in male ccRCC patients. Log-rank test was for **A**, **B**, **D** and **E**, while Likelihood ratio test was for Cox analyses in **C**, **F** and **G**

Higher DKC1 mRNA levels have been shown to be associated with shorter patient survival in the TCGA cohort of ccRCCs [23, 32], and therefore, we first performed the same analysis to confirm these results. Clinical data of these patients are listed in Additional file 1: Table S3. Patients were categorized into high and low groups using a median DKC1 mRNA value in tumors as the cutoff point. Indeed, patients in the high DKC1 group exhibited significantly shorter overall survival (OS) and progression-free survival (PFS) (Fig. 2A). We further determined the impact of sex on OS and PFS. As shown in Fig. 2B, female patients had longer PFS than male ones, while there was no difference in OS between them. Univariate COX regression analyses showed that higher DKC1 expression, advanced stages and grades were all associated with poor PFS, whereas female sex contributed to longer PFS (Fig. 2C). High DKC1 expression, advanced stages and grades predicted significantly shorter PFS independently, whereas female sex remained a variable for favorable PFS as determined using Multivariate analyses (Fig. 2C).

Having obtained the results above, we analyzed the impact of DKC1 expression on female and male patient survival separately. For female patients, significantly shorter OS and PFS was observed in the DKC1-high group (Fig. 2D), whereas OS and PFS in male patients were not affected by DKC1 expression (Fig. 2E). The median OS was not available (NA) (95% CI 79.5–NA months) for the DKC1-low group while 57.1 months (95% CI 42.3–92.1 months) for the high group in female patients (Low vs High: HR = 0.47; 95% CI 0.28–0.78, *P* = 0.0023). The HR for median PFS between the DKC1-low and high groups was 0.42 (95% CI 0.24–0.82, *P* = 0.0065). In contrast, median OS and PFS were not significantly different between DKC1-low and high groups in male patients (Low vs High OS: HR = 0.77, 95% CI 0.53–1.11, *P* = 0.1656; PFS: HR = 0.82, 95% CI 0.56–1.18, *P* = 0.2802). The combination of both sex and DKC1 expression revealed that female patients with DKC1-low tumors had the longest OS and PFS, especially for PFS (Additional file 1: Fig. S1). Univariate analyses of female patients revealed similar association between poor PFS and higher DKC1 expression, advanced stages and grades, as observed in the whole cohort of ccRCCs, and their independent impacts on PFS were further demonstrated by Multivariate COX regression analyses (Fig. 2F). For male patients, advanced stages and grades but not DKC1 expression predicted shorter PFS, as analyzed using a univariate analysis (Fig. 2G).

### Differences in proliferation, stemness, epithelial–mesenchymal transition (EMT) and genomic alterations between DKC1-low and high ccRCC tumors

**Fig. 3:**
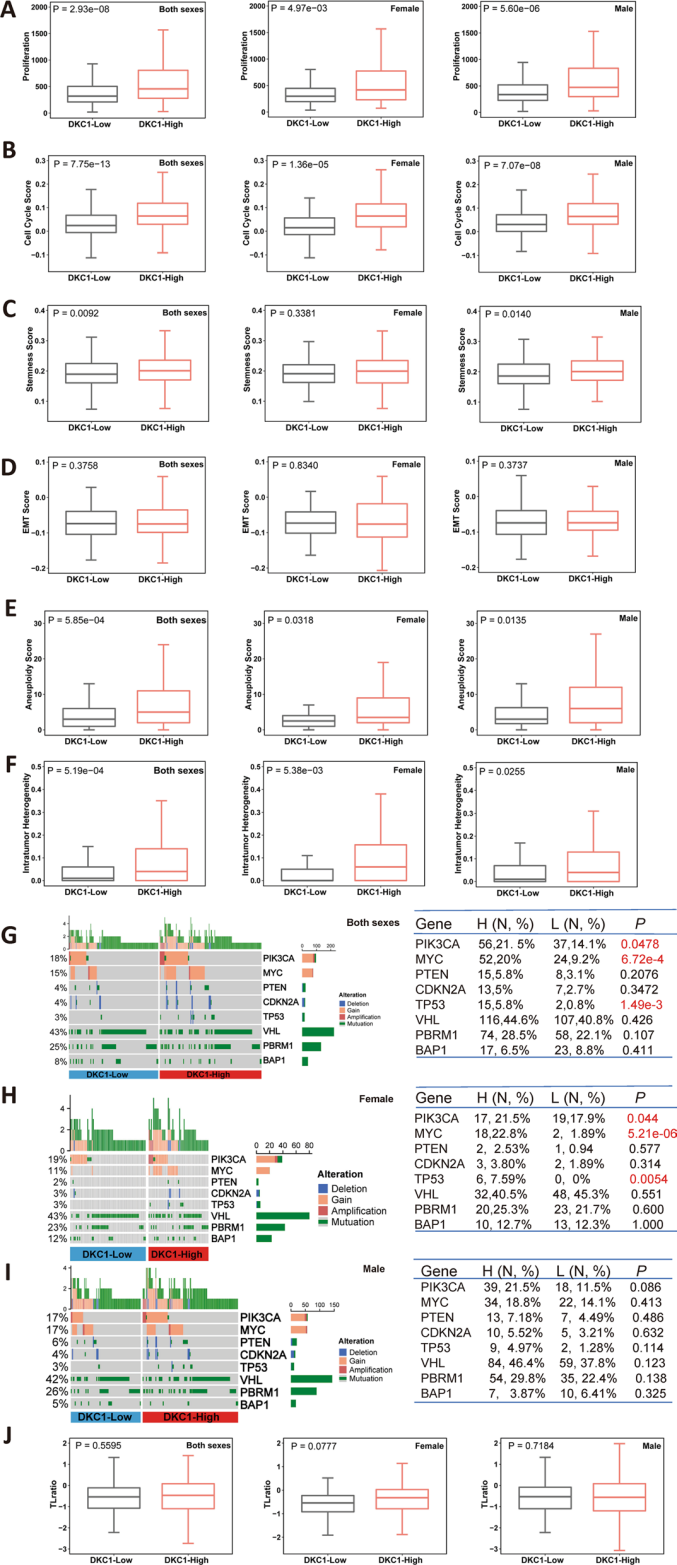
Differences in proliferation, phenotypes, genomic alterations and telomere length between DKC1-high and low ccRCC tumors. The analysis was performed on the total of 530 patients (both sexes), female and male patients in the TCGA ccRCC cohort, respectively. **A** and **B** Proliferation as determined using Ki-67 expression and cell cycle ssGSEA score, respectively. **C** Cancer stemness. **D** EMT. **E** Aneuploidy score. **F** Intratumor heterogeneity score. **G**–**I** Alterations in ccRCC-specific and non-specific genes in the total, female and male patients. **J** Telomere length. Wilcox sum test was used for **A**–**F** and **J**, while Fisher’s exact test was for **G**–**I**

We then sought to probe potential mechanisms underlying the DKC1 impact on patient survival. TERC was one of the key DKC1 targets, but its expression data were largely unavailable in the TCGA ccRCC cohort, and it was impossible to examine their relationship. Nevertheless, we compared important phenotypes (proliferation/cell cycle, stemness and EMT), and global and specific genomic alterations between DKC1-low and high ccRCC tumors. For proliferation analyses, Ki-67 was first used as the specific biomarker, and the DKC1-high tumors expressed significantly higher levels of Ki-67 independently of sex (DKC1-high vs low: female + male: *P* = 2.93E-08; female only: *P* = 4.97E-03; male only: *P* = 5.60E-06) (Fig. 3A). Then, the cell cycle score based on ssGSEA was evaluated and similar results were obtained (DKC1-high vs low: female + male: *P* = 7.75E-13; female only: *P* = 1.36E-05, male only: *P* = 7.07E-08) (Fig. 3B). Stem cell phenotype analyses showed significantly increased stemness in the DKC1-high groups from all the patients (*P* = 0.009) and male ones (*P* = 0.014) but not female patients (*P* = 0.338) (Fig. 3C). There were no differences in EMT between DKC1-high and low groups (Fig. 3D).

Genomic alterations were then compared between DKC1-low and high tumors. First, we made comparisons of the global aberrations: (1) Aneuploidy. A higher aneuploidy score was observed in the DKC1-high groups independently of gender (DKC1-high vs low: female + male: *P* = 5.85E-04; female only: *P* = 0.032, male only: *P* = 0.014) (Fig. 3E); (2) Intratumor heterogeneity. Similar results were obtained (DKC1-high vs low: female + male: *P* = 5.19E-04; female only: *P* = 0.0054, male only: *P* = 0.026) (Fig. 3F); (3) Tumor mutation burden (TMB). There were no significant differences in TMB (Additional file 1: Fig. S2). Second, we specifically analyzed frequently mutated ccRCC-related genes including *VHL*, *PBRM1* and *BAP1*. There were no differences in *VHL*, *PBRM1* and *BAP1* mutations between two groups in either female, male, or all patients (Fig. 3G–I). Third, since Ki-67 expression and cell cycle scores were robustly higher in the DKC1-high group, we analyzed alterations in proliferation-related oncogenes or tumor suppressors, including *PIK3CA*, *PTEN*, *TP53*, *MYC* and *CDKN2A*. Significantly higher frequencies of *PIK3CA* amplification/mutation, *MYC* amplification and *TP53* mutations were all observed in DKC1-high group (female + male) (Fig. 3G–I). The separate analyses showed that all these increased frequencies occurred in only female DKC1-high group (*P* = 0.005) but not male one (*P* = 0.114) (Fig. 3G–I). Finally, we compared telomere length between DKC1-high and low groups, and did not observe significant differences in either female, male or both (Fig. 3J).

### DKC1 as a predictor for Sunitinib response in female but not male patients

**Fig. 4:**
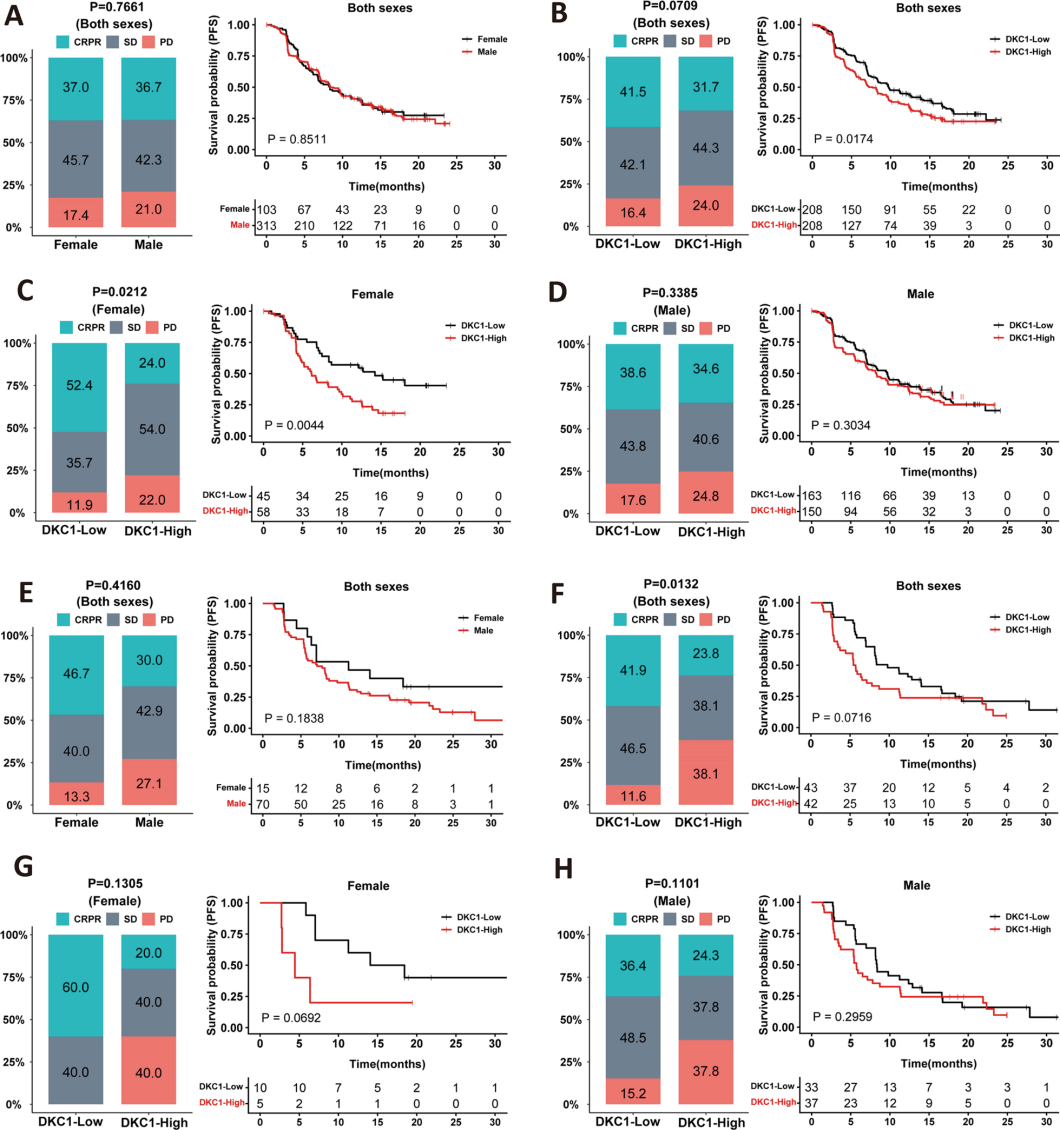
DKC1 as a predictor for Sunitinib response in female but not male ccRCC patients. The analyses were performed on IMmotion 151 (Discovery cohort **A**–**D**) and IMmotion150 (validation cohort, **E**–**H**) treated with Sunitinib. IMmotion 151 cohort: (**A**) differences in Sunitinib response (left) and PFS (right) between female and male ccRCC patients. (**B**) Differences in Sunitinib response (left) and PFS (right) between DKC1-high and low groups of ccRCC patients (both sexes). **C** Differences in Sunitinib response (left) and PFS (right) between DKC1-high and low groups in female patients. **D** Differences in Sunitinib response (left) and PFS (right) between DKC1-high and low groups in male patients. IMmotion 150 cohort. **E** Differences in Sunitinib response (left) and PFS (right) between female and male ccRCC patients. **F** Differences in Sunitinib response (left) and PFS (right) between DKC1-high and low groups of ccRCC patients. **G** Differences in Sunitinib response (left) and PFS (right) between DKC1-high and low groups in female patients. **H** Differences in Sunitinib response (left) and PFS (right) between DKC1-high and low groups in male patients. CRPR: complete and partial remission; SD: stable disease; PD: progressive disease. Left panels: Fisher’s exact test and right panels: log-rank test

We next sought to determine whether DKC1 expression was associated with Sunitinib response by analyzing IMmotion 151 cohort ccRCC patients. A total of 416 patients were treated with Sunitinib, among which were 103 females and 313 males. Clinical data of these patients are listed in Additional file 1: Table S4. Patient responses to Sunitinib were categorized into complete or partial remission (CR/PR), stable disease (SD) and progressive disease (PD) (Their definition is provided in the Additional file 1: Table S6). The Sunitinib efficacy was first compared between female and male patients, and no differences in PFS and response rates were observed (Fig. 4A). The impact of DKC1 on Sunitinib efficacy was then assessed. The median DKC1 expression value in tumors was used as a cutoff to divide patients into DKC1-high and low groups. The analysis of all 416 patients showed that CR/PR, SD and PD in the DKC1-high group were 31.7%, 44.3% and 24%, respectively, while in the DKC1-low group were 41.5%, 42.1% and 16.4, respectively. (Fig. 4B). The difference between DKC1-high and low groups was close to but did not reach statistical significance (*P* = 0.071). Nevertheless, patients in the DKC1-low group had longer median PFS compared to those in the DKC1-high group [high vs low: 7.7 vs 9.7 months, HR = 1.33 (95% CI 1.05–1.68), *P* = 0.017]. The separate analysis was further performed on female and male patients, respectively. For female patients treated with Sunitinib, the DKC1-low group had > twofold CR/PR rates than did high group (52.4% vs 24.0%), while PD rates were 11.9% and 22.0%, respectively (*P* = 0.021) (Fig. 4C). Consistently, median PFS was 14.2 and 6.1 months for DKC1-low and high groups, respectively [HR = 2.01 (95% CI 1.25–3.23), *P* = 0.004]. On the other hand, there were no significant differences in response rates (*P* = 0.338) and PFS (*P* = 0.303) between DKC1-high and low groups for male patients (Fig. 4D).

IMmotion 150 cohort of ccRCC patients were then analyzed as a validation set. Male and female patients treated with Sunitinib in this cohort were 70 and 15, respectively. The clinical information is summarized in Additional file 1: Table S5. There was no significant difference in response between female and male patients (Fig. 4E). The response rates of all 85 patients were 41.9%, 46.5% and 11.6% for CR/PR, SD and PD in the DKC1-low group, whereas 23.8%, 38.1% and 38.1% for CR/PR, SD and PD in the DKC1-high group, respectively (*P* = 0.013) (Fig. 4F). Median PFS was 11.3 and 7.1 months for DKC1-low and high groups, respectively (HR = 1.53 (95% CI 0.95–2.49, *P* = 0.072). The difference was not statistically significant. Separate analyses showed that the DKC1-high group in females exhibited reduced response rates and shorter PFS but without statistical differences compared to the DKC1-low group, which was likely due to a very small number of female patients (Fig. 4G). Male patients did not display DKC1-dependent response and survival (Fig. 4H).

### Association of TERC with DKC1 and Sunitinib response

**Fig. 5:**
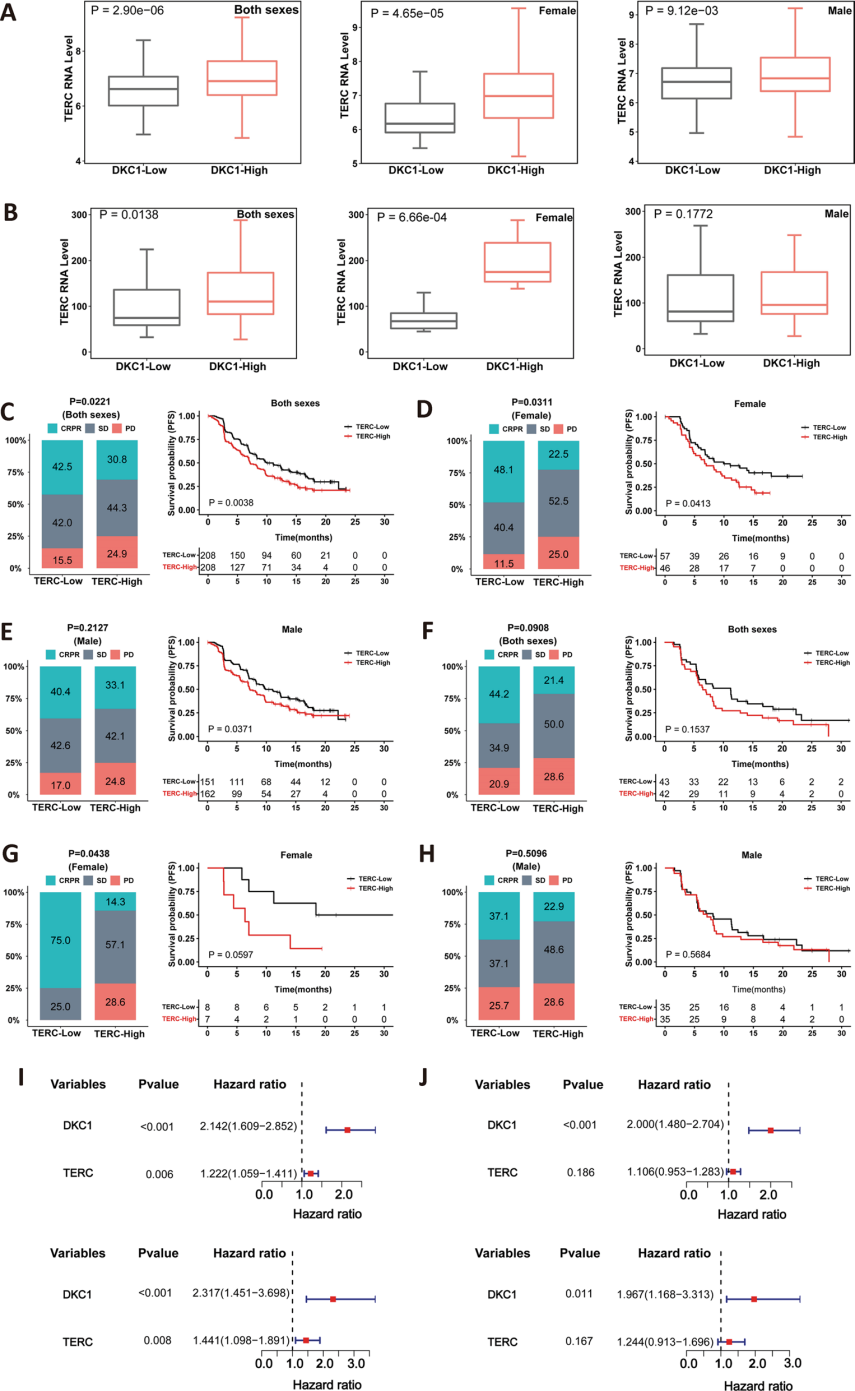
Concomitant expression of DKC1 and TERC and their impacts on Sunitinib response. The analyses were carried out on IMmotion 151 and IMmotion150 cohorts of ccRCC patients treated with Sunitinib. **A** and **B** Differences in TERC expression between DKC1-low and high groups in IMmotion151 (**A**) and IMmotion150 (**B**) cohorts. Left: all patients (both sexes); middle: female patients only and right: male patients only. **C**–**E** Differences in Sunitinib response and PFS between TERC-low and high groups in IMmotion151 cohort. (C) All patients (both sexes). **D** Female patients. **E** Male patients. **F**–**H** Differences in Sunitinib response and PFS between TERC-low and high groups in IMmotion150 cohort. (F) All patients (both sexes). **G** Female patients. **H** Male patients. **I** and **J** The evaluation of DKC1 and TERC as independent predictors for Sunitinib response and PFS. Univariate and multivariate COX regression analyses were performed on all patients (both sexes) (**I**) and female patients (**J**) from IMmotion151 cohort, respectively. CRPR: complete and partial response; SD: stable disease; PD: progressive disease. Panels A and B were evaluated using Wilcox sum test, while Fisher’s exact test was for **C**–**H**

Because DKC1 is fundamental to TERC stability and telomerase activity, we assessed whether DKC1 effect was attributable to TERC expression. Analyses of all 416 tumors from the IMmotion 151 ccRCC cohort revealed that TERC levels were robustly higher in DKC1-high than low groups (high vs low, *P* = 2.90E-06), and such scenarios occurred more robustly in female (*P* = 4.65E-05) than in male (*P* = 0.010) patients (Fig. 5A). For 85 ccRCC patients treated with Sunitinib in the IMmotion 150 cohort, the DKC1-high group exhibited significantly increased TERC expression (high vs low, *P* = 0.014); however, a highly significant increase was seen in female (*P* = 0.0007) but not male (*P* = 0.18) patients (Fig. 5B). These results were largely consistent with the observation from the IMmotion 151 cohort.

We then analyzed the impact of TERC expression on patient response to Sunitinib and survival. The patients in the IMmotion 151 cohort were divided into TERC-high and low groups using a median expression value as the cutoff point. CR/PR, SD and PD were 42.5%, 42.0% and 15.5% in the TERC-low group, while 30.8%, 44.3% and 24.9% in the TERC-high group, respectively (*P* = 0.022) (Fig. 5C). Female patients in the TERC-low group had > twofold CR/PR, while < 50% of PD compared with those in the high group (*P* = 0.031), and consistently, patient PFS in the low group was significantly longer (*P* = 0.041) (Fig. 5D). For male patients, the TERC-low group also displayed more CR/PR and less PD rates than did the TERC-high one, but the difference was not statistically significant (*P* = 0.213). Nevertheless, low TERC expression was beneficial to longer PFS in male patients (*P* = 0.037) (Fig. 5E).

For validation, the IMmotion 150 cohort was analyzed as same as above. CR/PR patients were > twofold more in the TERC-low than high group (44.2% vs 21.4%), but the difference did not reach a significant level (*P* = 0.091) (Fig. 5F); there was no difference in PFS between two groups either (*P* = 0.154) (Fig. 5F). CR/PR was observed in 75% of female patients in the TERC-low group without PD (*P* = 0.044), and PFS in this group was longer with a border line *P* value (*P* = 0.060) (Fig. 5G). For male patients, the TERC-low group displayed a higher CR/PR rate (low vs high: 37.1% vs 22.9%), but without a statistical difference (*P* = 0.510), and this was also the case in patient PFS (*P* = 0.568) (Fig. 5H).

As DKC1 is required for TERC expression, we further determined whether the observed TERC impact was dependent on DKC1. Sunitinib response rates and PFS inversely associated with both DKC1 and TERC levels were only observed in the female and all (female + male) patients from the IMmotion 151 cohort, and thus univariate and multivariate COX regression analyses were performed on them. As expected, univariate analyses showed that DKC1 and TERC were both associated with shorter PFS, while only DKC1 remained as a significant predictor in the female patients, as assessed using multivariate COX regression analyses (Fig. 5I). This was also the case in the whole cohort (Fig. 5J). Therefore, these results suggest that DKC1 rather than TERC independently predicts Sunitinib response and PFS.

### Association of DKC1 expression with the angiogenesis signature in ccRCC

**Fig. 6:**
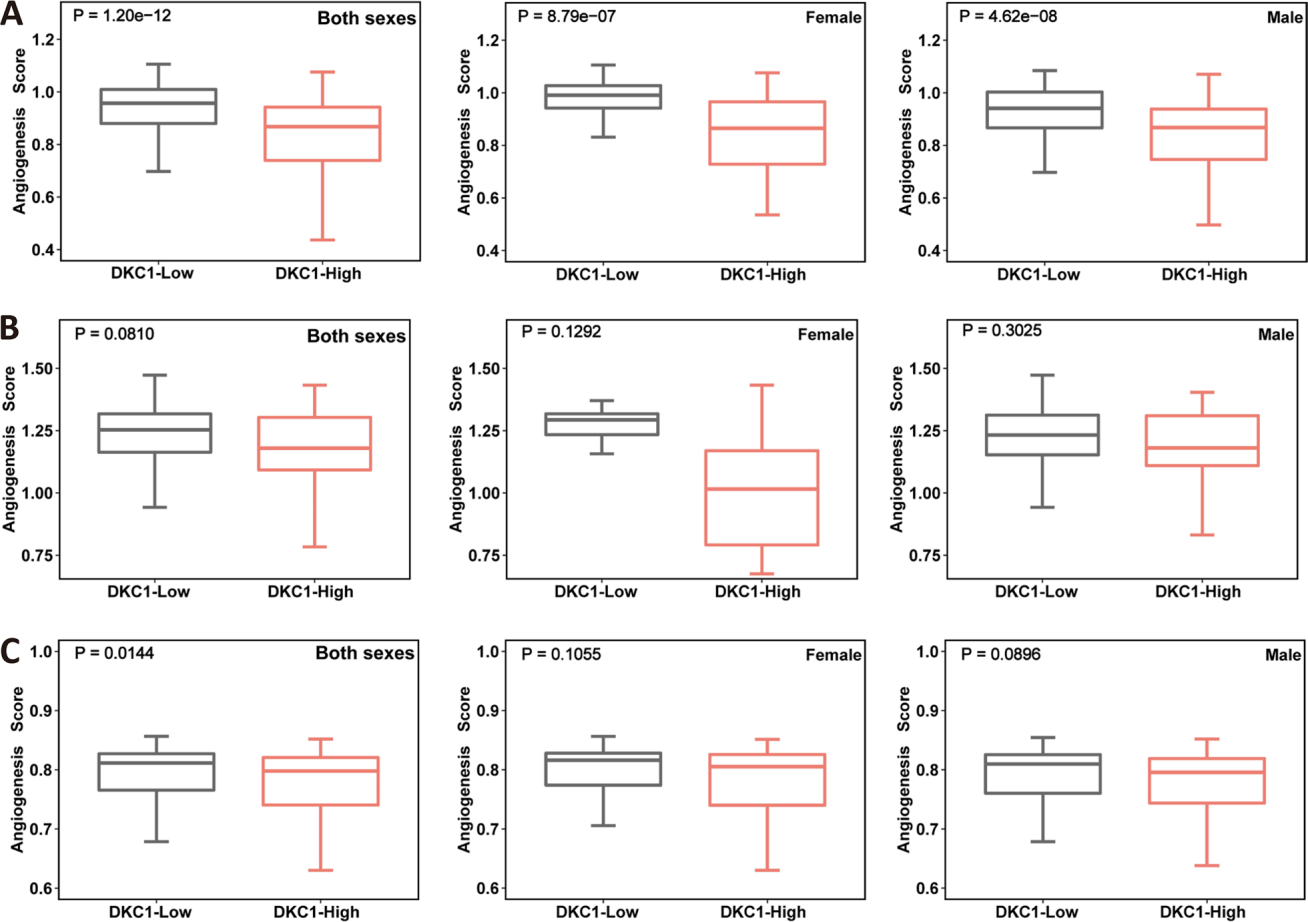
Poor angiogenesis in DKC1-high ccRCC tumors. Angiogenesis scores are calculated as described in Methods. **A** IMmotion151 cohort analyses. Left: all patients (both sexes); middle: female patients; right: male patients. **B** IMmotion150 cohort analyses. Left: all patients (both sexes); middle: female patients; right: male patients. **C** TCGA cohort analyses. Left: all patients (both sexes); middle: female patients; right: male patients. Wilcox sum test was used

We then sought to determine whether other aberrant signaling pathways contributed to the DKC1-mediated unfavorable Sunitinib response. Because the poor angiogenesis has been characterized as a predictor for Sunitinib resistance and shorter PFS [11], we compared its difference between DKC1-high and low groups. In the IMmotion 151 ccRCC patients treated with Sunitinib, the angiogenesis score was significantly reduced in the DKC1-high group compared to the low one (high vs low, *P* = 1.20E-12) (Fig. 6A, left), and the same phenomena were observed in both female (*P* = 8.79E-07) (Fig. 6A, middle) and male (*P* = 4.62E-08) patients (Fig. 6A, right). However, the analysis of 85 ccRCC patients, derived from the IMmotion 150 cohort (Sunitinib-treated), did not show significant differences in angiogenesis score between DKC1-high and low groups (*P* = 0.081), which was also the case in female (*P* = 0.129) and male (*P* = 0.303) patients (Fig. 6B). Given such inconsistent findings, we further analyzed the TCGA cohort. The lower angiogenesis score was observed in the DKC1-high group (*P* = 0.014), but the difference disappeared in the separate analysis of female (*P* = 0.106) and male (*P* = 0.089) patients (Fig. 6C).

## Discussion

Many biomarkers have been identified to predict ccRCC survival and response to adjuvant systemic therapies of TKR inhibitors; however, ccRCC displays sex-bias in incidence, aggressiveness and outcomes or mortality, and it is thus important to define which of them serve as sex-specific predictors. In the study presented herein, we show that DKC1 and TERC are associated with PFS and response to Sunitinib in female ccRCC patients only, and the findings are expected to contribute to the improvement of personalized managements for ccRCC and better understanding of sex-biased ccRCC pathogenesis.

DKC1 is overexpressed in various types of cancer [23, 32–41, 50] and functionally, present in several nuclear complexes to exert its biological activities: (1) the H/ACA small nucleolar RNA–ribonucleoprotein complex (H/ACAsnoRNP); (2) the H/ACA small Cajal body ribonucleoprotein complex (H/ACAscaRNP); and (3) the telomerase complex [20, 21]. DKC1-mediated TERC stabilization for telomerase activity has been well-characterized [21]. TERC harbors a H/ACA box RNA domain through which it is pseudouridylated at position 307 of the uridine residual in a DKC1-dependent manner [22]. DKC1 deficiency causes the X-linked dyskeratosis congenita disease (DC) featured with telomere pathologies [24, 25], while its overexpression enhances telomerase activity [51]. On the other hand, as the H/ACA sno/scaRNP catalytical component, DKC1 pseudouridylates many different RNA molecules (rRNAs, snRNAs, ncRNAs and mRNAs) to regulate ribosome biogenesis, cellular RNA splicing and translation, thereby actively participating in physiological and pathological processes [21]. Little has been known about these telomerase-independent effects of DKC1, and their roles in oncogenesis are poorly understood as well. The study by Yoon et al. [52] showed that DKC1 defects impaired translation from internal ribosome entry sites of specific cellular mRNAs, and these included XIAP and BCL2 that protected cells from apoptosis. In another study, DKC1 depletion induced proliferation arrest of neuroblastoma cells via TP53-dependent and independent pathways [35]. Mechanistically, DKC1 inhibition leads to destabilization of H/ACA snoRNAs and consequent disruption of ribosome biogenesis, thereby triggering a ribosomal stress response [35]. In those DKC1-depleted cells, cell cycle arrest was not rescued by TERC, which indicated that the observed DKC1 effect was telomerase or telomere length independent. More recently, DKC1 was found to promote proliferation, survival, invasion and metastasis of colorectal and hepatocellular cancer cells by enhancing HIF-1α expression and antioxidative effect, respectively [33, 39]. All these findings collectively suggest that DKC1 contributes to cancer development and progression in telomerase-dependent and independent manners.

Recent data have revealed that genomic alterations in cancer-related genes are sex-different in most kinds of cancer, which is also the case in ccRCC [14], but underlying mechanisms are elusive. The strong correlation between DKC1-high expression and altered PIK3CA, MYC and TP53 in female ccRCC patients suggests a role for DKC1 in sex-biased genomic aberrations. In addition, much attention has long been paid on X chromosome inactivation (XCI) skewing or escape and sex hormone signalings as potential mechanisms behind sex-differences in cancer [15, 16]. *DKC1* gene is located within Xq28, and one of its alleles is normally silent via XCI [25]. Interestingly, female *DKC1* mutation carriers in general do not manifest typical DC characteristics, largely due to the wild-type allele escape from XCI [25]. XCI escape aberrantly occurs in oncogenesis [15], and such mechanism may drive DKC1 dysregulation in female ccRCCs. Given its critical role in telomerase function and other carcinogenic processes, DKC1 dysregulation might promote ccRCC aggressiveness. Regarding sex hormone pathways, androgen receptor (AR) was shown to promote distant ccRCC metastasis and aggressiveness in male patients [16, 19]. In melanoma patients, AR signaling induction highly impairs efficacy of BRAF/MEK targeted therapy [53], which provides the explanation of why female patients display significantly higher response rates. The analysis results of IMmotion 151 and 150 cohorts showed no difference in Sunitinib efficacy between female and male ccRCC patients. Thus, different from melanoma, mechanisms underlying sex-specific effects in ccRCC are more complicated, and likely involved in interactions among many more signaling pathways including DKC1.

It is currently unclear how DKC1 specifically exerts its negative impact on PFS and response to Sunitinib in female ccRCC patients. Our findings provide potential explanations as follows: (1) the difference in TERC expression between DKC1-high and low groups is much more marked in female than in male patients. Increased TERC expression not only enhances telomerase activity to lengthen telomeres, but also promotes cancer aggressiveness independently of the canonical telomerase function [54]. For instance, TERC acts as a transcription co-factor to stimulate transcription of NF-KB target genes [55], while DKC1 inhibition impairs growth and invasion of ccRCC-derived cells via the NF-KB cascade [32]. Moreover, a hyperactive NF-KB pathway plays a part in cancer progression and Sunitinib resistance [56]. Of note, in the multivariate analysis, DKC1 rather than TERC served as an independent predictor for Sunitinib efficacy, suggesting the TERC impact resulting from DKC1. (2) For female patients, the tumor suppressor *TP53* mutation occurs predominantly in the DKC1-high group, while there are no differences between male DKC1-high and low patients. *TP53* mutations are known to be associated with inferior survival and poor Sunitinib response [8, 57, 58]. (3) The female DKC1-high group has significantly higher frequencies of *PIK3CA* amplification/mutation and *MYC* amplification, which might contribute to hyper-proliferation, survival and aggressive phenotypes [59], thereby inducing Sunitinib resistance. (4) Poor angiogenesis and enhanced cell cycle/proliferation, established biomarkers to predict Sunitinib resistance [11, 60], are present in the DKC1-high group, which lead to impaired Sunitinib response. However, these same differences occurred in both female and male patients and were unable to explain unequal efficacies of the DKC1-low groups between them. Further investigations are required to answer this question.

*DKC1* is a defined MYC target gene. Intriguingly, the *MYC* gene amplification occurred in almost one-quarter of DKC1-high group while only < 2% of DKC1-low one in female patients. Such a difference was not observed in male patients. The *MYC* gene aberration may be an additional mechanism behind DKC1 upregulation in tumors from female patients.

### Perspectives and significance

Significant upregulation of DKC1 expression occurs in ccRCC tumors. High DKC1 expression predicts shorter PFS independently of stages and grades in female but not male patients. Importantly, high DKC1 expression confers poor response to Sunitinib and shorter PFS in female patients. Collectively, DKC1 serves as a female-specific predictor for outcomes and Sunitinib treatment efficacy in ccRCC independently. The present findings are expected to provide new insights into the sex-biased pathogenesis and to benefit to personalized interventions in ccRCC.

## Supplementary Information

**Additional file 1: Table S1.** Characteristic of Qilu Cohort of ccRCC patients for RNA sequencing. **Table S2.** Characteristic of Qilu ccRCC Cohort for qPCR assays. **Table S3.** Characteristic of TCGA ccRCC Cohort. **Table S4.** Characteristic of IMmotion151 Cohort treated with Sunitinib. **Table S5.** Characteristic of IMmotion150 Cohort treated with Sunitinib. Table S6: Definition of complete remission, partial remission, stable diseaseand progressive disease. **Figure S1.** Effect of the combined sex and DKC1 expression on overall and progression-free survivalin the TCGA cohort of ccRCC patients. **Figure S2.** Tumor mutation burdenin DKC1-low and high tumors from the TCGA ccRCC cohort.

## Data Availability

Source data downloaded from public databases are provided with this paper. Data from the IMmotion150 and151 trial were downloaded from European Genome–Phenome Archive (EGA) under accession number EGAS00001002928 and EGAC00001001813 with EGA approval. Any additional information required to reanalyze the data reported in this paper is available from the corresponding authors upon reasonable request. The RNA sequencing data from 10 ccRCC patients were deposited in the GEO database (GSE217386).

## Abbreviations

AR: Androgen receptor
ccRCC: Clear cell renal cell carcinoma
CR/PR: Complete/partial remission
CNA: Copy number alteration
CSF1R: Colony stimulating factor-1 receptor
DKC1: Dyskerin
FLT3: Fms-like tyrosine kinase 3
NT: Noncancerous tissues
PD: Progressive disease
PDGFRs: Platelet-derived growth factor receptors
SD: Stable disease
TERC: Telomerase RNA component
TERT: Telomerase reverse transcriptase
TKR: Tyrosine-kinase receptor
VEGFRs: Vascular endothelial growth factor receptors
XCI: X chromosome inactivation
