# Supplementary material for: Dyskerin and telomerase RNA component are sex-differentially associated with outcomes and Sunitinib response in patients with clear cell renal cell carcinoma
Source: Biol Sex Differ. 2023 Jul 11;14:46. doi: 10.1186/s13293-023-00526-7 (PMC10334619; doi:10.1186/s13293-023-00526-7)
Supplement: Supplementary file 1 — Additional file 1: Table S1. Characteristic of Qilu Cohort of ccRCC patients for RNA sequencing. Table S2. Characteristic of Qilu ccRCC Cohort for qPCR assays. Table S3. Characteristic of TCGA ccRCC Cohort. Table S4. Characteristic of IMmotion151 Cohort treated with Sunitinib. Table S5. Characteristic of IMmotion150 Cohort treated with Sunitinib. Table S6: Definition of complete remission, partial remission, stable diseaseand progressive disease. Figure S1. Effect of the combined sex and DKC1 expression on overall and progression-free survivalin the TCGA cohort of ccRCC patients. Figure S2. Tumor mutation burdenin DKC1-low and high tumors from the TCGA ccRCC cohort. [file 13293_2023_526_MOESM1_ESM.pdf]

***Table S1**. Characteristic of Qilu Cohort of ccRCC patients for RNA sequencing*

Qilu Hospital Cohort	**[ALL]** *N=10*
Age, mean (SEM[a])	52.7 (10.3)
Sex, n (%)	
Female	3 (30.0%)
Male	7 (70.0%)
AJCC Stage[b], n (%)	
I	5 (50.0%)
II	1 (10.0%)
vgt III	4 (40.0%)
T, n (%)	
T1	5 (50.0%)
T2	1 (10.0%)
T3	4 (40.0%)
Lymph node metastasis, n (%)	
N0	9 (90.0%)
N1	1 (10.0%)
Grade, n (%)	
G1/G2	5 (50.0%)
G3/G4	5 (50.0%)
Tumor max diameter, n (%)	
<4	2 (20.0%)
4-7	4 (20.0%)
7-10	2 (40.0%)
>10	2 (20.0%)

[a]SEM, standard error of mean.

[b]Tumor AJCC stages according to the American Joint Committee on Cancer (AJCC) 7th edition.

Table S2. Characteristic of Qilu ccRCC Cohort for qPCR assays

	**Qilu Cohort**
	N=10
Age, mean (SEM[a])	52.7 (10.3)
Sex, *n (%)*	
Female	3 (30.0%)
Male	7 (70.0%)
AJCC Stage[b], *n (%)*	
I	5 (50.0%)
II	1 (10.0%)
III	4 (40.0%)
T, *n (%)*	
T1	5 (50.0%)
T2	1 (10.0%)
T3	4 (40.0%)
Lymph node metastasis, *n (%)*	
N0	9 (90.0%)
N1	1 (10.0%)
Grade, *n (%)*	
G1/G2	5 (50.0%)
G3/G4	5 (50.0%)
Tumor max diameter(cm), *n (%)*	
<4	2 (20.0%)
4-7	4 (20.0%)
7-10	2 (40.0%)
>10	2 (20.0%)

[a]SEM, standard error of mean.

[b]Tumor AJCC stages according to the American Joint Committee on Cancer (AJCC) 7th edition.

Table S3. Characteristic of TCGA ccRCC Cohort

	DKC1-Low	DKC1-High	Pvalue
	N=265	***N=265***	
Age, mean (SEM[a])	60.3 (12.1)	61.1 (12.2)	0.421
Sex, *n (%)*			0.023
Male	159 (60.0%)	185 (69.8%)	
Female	106 (40.0%)	80 (30.2%)	
T, *n (%)*			0.069
T1	142 (53.6%)	130 (49.1%)	
T2	35 (13.2%)	34 (12.8%)	
T3	87 (32.8%)	92 (34.7%)	
T4	1 (0.38%)	9 (3.40%)	
Lymph node metastasis, *n (%)*			0.424
N0	130 (95.6%)	110 (92.4%)	
N1	6 (4.41%)	9 (7.56%)	
Metastasis, *n (%)*			0.005
M0	220 (89.4%)	202 (79.8%)	
M1	26 (10.6%)	51 (20.2%)	
AJCC Stage[b], *n (%)*			0.025
I	140 (53.2%)	126 (47.7%)	
II	32 (12.2%)	25 (9.47%)	
III	63 (24.0%)	60 (22.7%)	
IV	28 (10.6%)	53 (20.1%)	
Grade, *n (%)*			0.027
G1	10 (3.85%)	4 (1.53%)	
G2	122 (46.9%)	106 (40.5%)	
G3	101 (38.8%)	105 (40.1%)	
G4	27 (10.4%)	47 (17.9%)	

[a]SEM, standard error of mean.

[b]Tumor AJCC stages according to the American Joint Committee on Cancer (AJCC) 7th edition.

Table S4. Characteristic of IMmotion151 Cohort treated with Sunitinib

	**DKC1-Low**	**DKC1-High**	**Pvalue**
	N=208	***N=208***	
Age, mean (SEM[a])	60.2 (9.35)	59.3 (10.5)	0.344
Sex, *n (%)*			0.173
Female	45 (21.6%)	58 (27.9%)	
Male	163 (78.4%)	150 (72.1%)	
Histology, *n (%)*			<0.001
ccRCC-nonSarcoma	189 (90.9%)	157 (75.8%)	
ccRCC-Sarcoma	17 (8.17%)	39 (18.8%)	
nonccRCC-Sarcoma	2 (0.96%)	11 (5.31%)	
IMDC[b], *n* (%)			<0.001
Favorable	59 (28.4%)	29 (13.9%)	
Intermediate	126 (60.6%)	127 (61.1%)	
Poor	23 (11.1%)	52 (25.0%)	
Liver Metastases, *n (%)*			0.057
no	178 (85.6%)	162 (77.9%)	
yes	30 (14.4%)	46 (22.1%)	
TMB, mean (SEM[a])	3.86 (2.16)	4.32 (2.31)	0.067
Response[c], *n (%)*			0.070
CR/PR	81 (41.5%)	58 (31.7%)	
PD	32 (16.4%)	44 (24.0%)	
SD	82 (42.1%)	81 (44.3%)	

[a]SEM, standard error of mean.

IMDC[b], International Metastatic Renal Cell Carcinoma Database Consortium Criteria

Response[c], CR, complete response;

PR, partial response;

SD, stable disease;

PD, progressive disease;

Table S5. Characteristic of IMmotion150 Cohort treated with Sunitinib

	**DKC1-Low**	**DKC1-High**	**Pvalue**
	N=43	***N=42***	
Age, mean (SEM[a])	60.2 (10.6)	60.5 (10.6)	0.908
Sex, *n (%)*			0.277
Female	10 (23.3%)	5 (11.9%)	
Male	33 (76.7%)	37 (88.1%)	
TMB, mean (SEM[a])	8.91 (3.15)	32.0 (118)	0.269
Metastasized, *n* (%)			
Yes	43 (100%)	42 (100%)	1
Response[b], *n (%)*			0.013
CR/PR	18 (41.9%)	10 (23.8%)	
PD	5 (11.6%)	16 (38.1%)	
SD	20 (46.5%)	16 (38.1%)	

[a]SEM, standard error of mean.

Response[b], CR, complete response;

PR, partial response;

SD, stable disease;

PD, progressive disease;

Table S6. The definition of complete remission (CR), partial remission (PR), stable disease (SD) and progressive disease (PD)*

CR: Disappearance of all target and non-target lesions and (if applicable) normalization of tumor marker level; or reduction in short axis of any pathological lymph nodes (whether target or non-target) to less than (<) 10 mm.

PR: At least a 30% decrease in the sum of diameters of target lesions, taking as reference the baseline sum of diameters; or persistence of one or more non-target lesion(s) and/or (if applicable) maintenance of tumor marker level above the normal limits.

SD: Neither sufficient shrinkage to qualify for PR nor sufficient growth to qualify for progressive disease, with no new lesions. This requires that the sum of the LDs of target lesions have a decrease of less than 30% or an increase of less than 20%, and last for a minimum of 6 weeks.

PD: At least a 20% increase in the sum of diameters of all target and new measurable lesions, taking as reference the smallest sum on study, including baseline, and an absolute increase of at least 5 mm.

*CR, PR, SD and PD in the Sunitinib-treated cohorts are based on Response Evaluation Criteria in Solid Tumors Version 1.1 (https://clinicaltrials.gov/ct2/show/NCT02420821?term=NCT02420821&draw=2&rank=1).

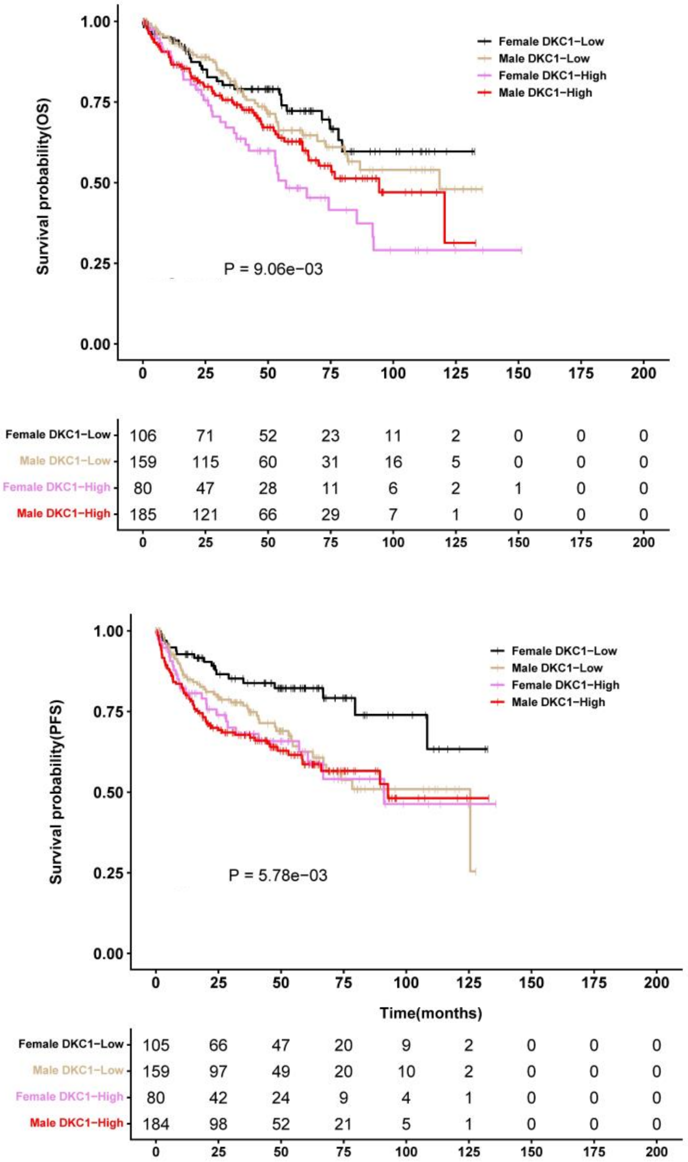

Figure S1. The analysis of the combined sex and DKC1 expression on overall and progression-free survival (OS and PFS) in the TCGA cohort of ccRCC patients. The patient numbers were 530 and 528 for OS and PFS analyses, respectively, and they were categorized into the following 4 groups: Female DKC1-low, male DKC1-low, female DKC1-high and male DKC1-high. The cut-off point for DKC1 expression was the median expression level. (A) OS and (B) PFS.

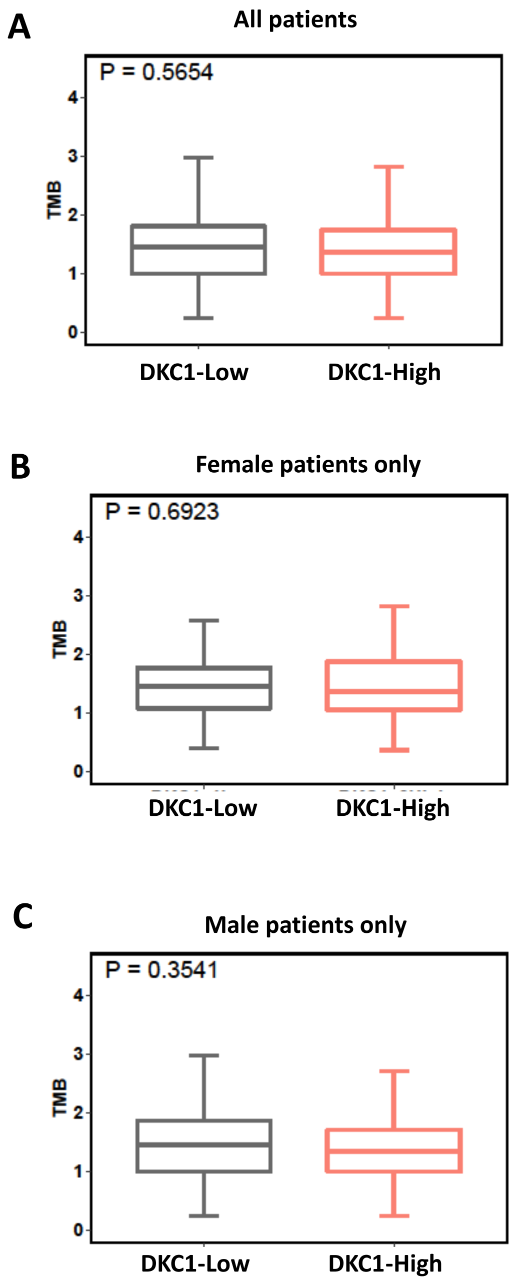

Figure S2: Tumor mutation burden (TMB) in DKC1-low and high tumors from the TCGA ccRCC cohort. The TMB analysis was performed on tumors from 530 patients in the TCGA ccRCC cohort and DKC1-low and high groups were classified based on median DKC1 mRNA levels in tumors as a cutoff point. (A) TMB in DKC1-low and high groups from all the patients. (B) TMB in female DKC1-low and high groups. (C) TMB in male DKC1-low and high groups.
